# 异基因造血干细胞移植治疗急性髓系白血病伴BCR::ABL1融合基因7例临床分析

**DOI:** 10.3760/cma.j.issn.0253-2727.2023.12.005

**Published:** 2023-12

**Authors:** 梦泽 郝, 小利 赵, 潇予 张, 圆圆 施, 明 龚, 利宁 张, 书连 陈, 嘉璘 魏, 祎 何, 四洲 冯, 明哲 韩, 尔烈 姜

**Affiliations:** 1 中国医学科学院血液病医院（中国医学科学院血液学研究所），实验血液学国家重点实验室，国家血液系统疾病临床医学研究中心，细胞生态海河实验室，天津 300020 State Key Laboratory of Experimental Hematology, National Clinical Research Center for Blood Diseases, Haihe Laboratory of Cell Ecosystem, Institute of Hematology & Blood Diseases Hospital, Chinese Academy of Medical Sciences & Peking Union Medical College, Tianjin 300020, China; 2 天津医学健康研究院，天津 301600 Tianjin Institutes of Health Science, Tianjin 301600, China

**Keywords:** 急性髓系白血病, 融合基因，BCR::ABL1, 异基因造血干细胞移植, Acute myeloid leukemia, Fusion gene, BCR::ABL1, Allogeneic hematopoietic stem cell transplantation

## Abstract

**目的:**

探讨异基因造血干细胞移植（allo-HSCT）治疗急性髓系白血病伴BCR::ABL1（AML伴BCR::ABL1）患者的疗效。

**方法:**

纳入2012年11月至2022年1月在中国医学科学院血液病医院诊断为AML伴BCR::ABL1并接受allo-HSCT的7例患者，回顾性分析其临床资料。

**结果:**

7例患者起病时中位年龄35（21～40）岁，4例（57.1％）白细胞增高。7例患者BCR::ABL1融合基因均为阳性且伴有NPM1、RUNX1、ASXL1、PHF6基因突变。7例患者均接受诱导化疗联合酪氨酸酶抑制剂（TKI）靶向治疗并桥接allo-HSCT，移植后6例患者继续行TKI维持治疗。移植前6例获形态学完全缓解（CR），4例获完全分子生物学缓解（CMR），allo-HSCT后其余3例也获得CMR。1例患者移植后因感染死亡，6例无复发存活，移植后3年总生存率为（80.0±17.9）％。

**结论:**

诱导化疗联合TKI治疗AML伴BCR::ABL1疗效较好，桥接allo-HSCT可进一步提高CMR，移植后TKI维持治疗可能有助于改善预后。

## Abstract (en)

**Objective:**

To explore the efficacy of allogeneic hematopoietic stem cell transplantation（allo-HSCT）in acute myeloid leukemia（AML）patients with BCR::ABL1 fusion.

**Methods:**

The clinical data of seven AML patients with BCR::ABL1 fusion from November 2012 to January 2022 were retrospectively analyzed, and their survival status was followed up.

**Results:**

The median age of patients at the time of diagnosis was 35 years. Four cases（57.1％）were diagnosed with high leukocyte counts. All cases were assayed as BCR::ABL1 positive and accompanied by four types of gene mutations（NPM1, RUNX1, ASXL1, PHF6）. Seven patients received tyrosine kinase inhibitor（TKI）combined with induction chemotherapy and bridged to allo-HSCT, and six patients received maintenance therapy with TKI. Before allo-HSCT, six patients achieved complete remission, and four patients achieved complete molecular remission（CMR）. After allo-HSCT, the three remaining cases also achieved CMR. All patients were in remission post-allo-HSCT. One case died of infection, and the remaining cases survived without relapse. The 3-year cumulative overall survival rate was（80.0±17.9）％.

**Conclusion:**

TKI combined with traditional chemotherapy could achieve a high response rate in AML patients with BCR::ABL1 fusion. In addition, allo-HSCT could enhance the molecular response rate. Maintenance therapy post-HSCT with TKI could improve prognosis.

急性髓系白血病伴BCR::ABL1（AML伴BCR::ABL1）是2016年世界卫生组织（WHO）造血与淋巴系统肿瘤分类中新增的暂定罕见肿瘤类型[1]。2022年第5版WHO造血淋巴肿瘤分类中关于AML伴BCR::ABL1的定义进一步明确[2]。不同于伴其他重现性细胞遗传学的AML，诊断该类疾病仍需要外周血或骨髓原始细胞≥20％[2]。依据2022欧洲白血病组（ELN）对白血病预后分组的建议，AML伴BCR::ABL1归于预后不良组[3]。在AML中，AML伴BCR::ABL1占比低于1.5％，属于罕见类型[4]。既往报道生存率较低，预后较差，尚无统一的治疗方案[5]–[8]。近年来，我中心采用异基因造血干细胞移植（allo-HSCT）治疗7例AML伴BCR::ABL1患者并取得良好疗效。

## 病例与方法

一、病例

纳入2012年11月至2022年1月在我中心诊断为AML伴BCR::ABL1的7例患者，其中6例为原发性AML伴BCR::ABL1，1例为既滤泡淋巴瘤病史的AML伴BCR::ABL1，诊断均符合WHO 2022的诊断标准。

二、检测方法

外周血及骨髓形态：经瑞氏-吉姆萨染色后显微镜下观察细胞形态。流式细胞术分析：依照本院常规流式细胞术规范，使用FACS Calibur型流式细胞仪进行活细胞检测。以SSC/CD45设门，抗体组合进行免疫分型分析。细胞遗传学分析：经培养瓶直接或24 h培养，按照常规染色体制备、R/G显带技术，镜下分析至少20个分裂象，染色体核型描述参照ISCN2009标准。分子生物学分析：按照TRIzol（美国Invitrogen公司产品）法提取总RNA并反转录cDNA，应用多重巢式PCR法检测相关融合基因，其中包括BCR::ABL1的定量分析。基因突变采用illumia二代测序（NGS）方法，对急性白血病相关基因突变进行检测。

三、预处理方案

7例患者中5例采用白消安/环磷酰胺/氟达拉滨/阿糖胞苷预处理，2例采用白消安/环磷酰胺/氟达拉滨/去甲氧柔红霉素。具体剂量：白消安3.2 mg·kg^−1^·d^−1^×3 d，环磷酰胺40 mg·kg^−1^·d^−1^×2 d，氟达拉滨30 mg·m^−2^·d^−1^×3 d，阿糖胞苷2 g·m^−2^·d^−1^×3 d，去甲氧柔红霉素10 mg·m^−2^·d^−1^×3 d。无关供者移植、单倍体移植加用兔抗人胸腺细胞免疫球蛋白2.5 mg·kg^−1^·d^−1^×4 d。

四、移植后主要并发症防治

采用环孢素A或他克莫司联合短疗程甲氨蝶呤±霉酚酸酯预防移植物抗宿主病（GVHD）。移植前予复方磺胺甲唑（1.92 g/d×5 d）预防卡氏肺孢子菌肺炎。移植后给予泊沙康唑预防真菌感染。

五、疗效评价

疗效评价参照血液病诊断及疗效标准（第四版）[9]。完全缓解（CR）：骨髓原始细胞<5％、外周血无原始细胞、无髓外白血病、外周血常规恢复；完全分子学缓解（CMR）：流式细胞术检测微小残留病（MRD）/ BCR::ABL1定量检测阴性。

六、随访

通过查阅门诊/住院病例和电话随访方式完成随访。总生存（OS）时间：造血干细胞回输至随访截止或死亡（任何原因）的时间。

七、统计学处理

应用SPSS 22.0软件分析数据。计量资料以“中位数（范围）”表示。生存率以Kaplan-Meier曲线进行描述。

## 结果

一、临床特征

7例患者确诊时中位年龄35（21～40）岁，其中男5例，女2例。FAB分型：M_5b_ 4例，M_2_ 1例，M_0_ 1例。起病时血常规（中位数）：WBC 113.9（6.8～282.6）×10^9^/L，PLT 77（10～424）×10^9^/L，HGB 105（66～148）g/L，4例患者嗜碱性粒细胞增多［中位数0.35（0.27～0.78）×10^9^/L］。7例患者异常细胞群免疫表型均为髓系抗原表达，5例强表达/表达干细胞抗原CD34。5例为BCR::ABL P210，1例为BCR::ABL P230，1例BCR::ABL P210和P190均为阳性。二代测序筛查发现4种一类基因突变：NPM1、RUNX1、ASXL1、PHF6。染色体核型分析：4例患者单纯伴t（9；22），其余3例合并其他核型异常。FISH检测：2例伴TP53缺失。7例患者起病时未行ABL区激酶突变检测，治疗期间检测均未发现激酶区突变。起病时3例患者无脾脏肿大，3例中度脾大，1例重度脾大。详见表1。

**表1:** 7例急性髓系白血病伴BCR::ABL1患者的临床特征

例号	性别	年龄（岁）	FAB分型	初诊血常规				骨髓原始细胞（%）	脾脏肿大	染色体核型	BCR:: ABL1	基因突变
				WBC（×10^9^/L）	嗜碱粒细胞（×10^9^/L）	PLT（×10^9^/L）	HGB（g/L）					
1	男	38	M_5b_	154.0	0.32	77	109	37.5	中度	46,XY,t(9;22;14)(q34;q11.2;p13)[20]	P210	RUNX1
2	男	26	M_5b_	282.6	0.78	43	105	67.5	中度	46,XY,t(9;22)(q34;q11.2)[4]	P190，P210	NPM1
3	女	35	M_0_	204.0	0.38	10	91	72.0	重度	47,XX,add(5)(q35),add(8)(q24),?del(9)(q21),del(15)(q22),add(17)(p12),der(22)t(9;22)(q34,q11),+mar,inc[20]	P210	无
4	男	21	NA	47.7	0.06	93	73	30.0	中度	52,XY,+8,+8,t(9;22)(q34;q11.2),+i(17)(q10),+19,+der(22)t(9;22)x2[20]	P230	PHF6
5	女	36	M_5b_	29.4	NA	217	118	35.0	无	+8，t(9;22)，i(17q)	P210	ASXL1RUNX1
6	男	32	M_5b_	6.8	0.05	424	66	57.5	无	46,XY,t(9;22)(q34.1;q11.2)[20]	P210	ASXL1
7	男	40	M_2_	113.9	NA	27	148	38.5	无	46，XY，t(9;22)(q34;q11)[20]	P210	NA

二、移植前治疗

7例患者诱导/再诱导化疗方案均以标准剂量阿糖胞苷（Ara-C）100～200 mg·m^−2^·d^−1^×7 d联合柔红霉素（DNR）60～90 mg·m^−2^·d^−1^或去甲氧柔红霉素（IDA）12 mg·m^−2^·d^−1^×3 d为基础，联合酪氨酸激酶抑制剂（TKI）靶向治疗。其中4例选用伊马替尼，例1因再诱导化疗后骨髓未缓解（NR）调整为达沙替尼，例3因2个疗程巩固化疗后P210仍阳性且数值升高而调整为达沙替尼；3例选用达沙替尼，例6因4个疗程巩固化疗后P210持续阳性而调整为奥雷巴替尼。诱导化疗后5例（71.4％）达到CR，1例部分缓解（PR），1例NR。经历3～7个疗程化疗后4例患者获得CMR（BCR::ABL1阴性）。诱导化疗过程中，例2出现反复发热、肾功能异常、少尿、血氧饱和度下降，结合其起病高白细胞状态，考虑肿瘤溶解综合征。余3例高白细胞患者在诱导化疗中未出现类似症状。详见表2。

**表2:** 7例急性髓系白血病伴BCR::ABL1患者的治疗及转归

例号	诱导化疗方案	TKI	再诱导化疗方案	巩固化疗	移植前评价	移植模式	移植后维持治疗	移植后3个月评价	OS（月）	结局
1	IA	伊马替尼	IA+伊马替尼，HAG+达沙替尼	1疗程+达沙替尼	NR	haplo-HSCT	达沙替尼	CMR	14	死亡
2	DA	伊马替尼	无	3疗程+伊马替尼	CMR	URD-HSCT	伊马替尼	CMR	82	生存
3	DOAP	伊马替尼	AAG+伊马替尼	3疗程+达沙替尼	CR MRD阳性	MSD-HSCT	达沙替尼	CMR	70	生存
4	HA	达沙替尼	无	6疗程+达沙替尼	CMR	haplo-HSCT	达沙替尼→氟马替尼	CMR	32	生存
5	DA	达沙替尼	无	2疗程+达沙替尼	CMR	haplo-HSCT	达沙替尼→伊马替尼	CMR	11	生存
6	DA	达沙替尼	无	5疗程+达沙替尼 1疗程+奥雷巴替尼	CR MRD阳性	MSD-HSCT	奥雷巴替尼	CMR	6	生存
7	HAM	伊马替尼	无	2疗程+伊马替尼	CMR	MSD-HSCT	无	CMR	120	生存

注 IA：阿糖胞苷＋去甲氧柔红霉素；DA：柔红霉素+阿糖胞苷；HA：高三尖杉酯碱+阿糖胞苷；HAM：高三尖杉酯碱+阿糖胞苷+米托蒽醌；HAG：高三尖杉酯碱+阿糖胞苷+重组人粒细胞集落刺激因子；AAG：阿糖胞苷+阿克拉霉素+粒细胞集落刺激因子；DOAP：柔红霉素+阿糖胞苷+长春地辛+泼尼松片；TKI：酪氨酸激酶抑制剂；haplo-HSCT：单倍体造血干细胞移植；MSD-HSCT：同胞全相合造血干细胞移植；URD-HSCT：无关供者造血干细胞移植；NR：未缓解；CR：形态学缓解；MRD：微小残留病；CMR：分子学缓解；OS：总生存

三、移植方案及维持治疗

7例患者化疗后均桥接allo-HSCT，移植前4例达CMR，2例达形态学缓解但BCR::ABL1阳性，1例NR（表2）。3例行单倍体造血干细胞，3例行同胞全相合造血干细胞移植，1例行全相合无关供者造血干细胞移植。单个核细胞（MNC）回输量为9.18（7.00～14.61）×10^8^/kg，CD34^+^细胞回输量为2.99（2.68～11.48）×10^6^/kg。7例患者均植活，中位粒细胞植入时间为12（11～20）d，中位血小板植入时间为13（9～30）d。移植前NR的例1及微小残留病（MRD）阳性的例3、例6均在移植后3月获得CMR（表2）。移植后6例患者行TKI维持治疗，维持治疗起始时间为移植后1～6个月，血小板计数持续≥80×10^9^/L时用药。例1移植前NR，移植后1个月BCR::ABL1P210仍为阳性，遂抢先加用达沙替尼，后因急性GVHD改为间断口服。例4、例5发生多浆膜腔积液、全血细胞下降不能耐受达沙替尼维持治疗，分别调整为氟马替尼、伊马替尼。6例患者中例1死亡，其余5例存活患者维持治疗时间为1～1.5年。

四、移植相关并发症

4例患者发生急性GVHD（2例为Ⅱ～Ⅳ度），4例发生慢性GHVD，1例在移植后17个月出现血小板减少，3例发生巨细胞病毒血症，1例患者出现出血性膀胱炎。

五、预后

至随访截止，6例患者存活，原发病均持续CMR状态，1例（例1）死亡，3年OS率为（80.0±17.9）％。例1移植前疾病状态为NR，行单倍体造血干细胞移植后出现Ⅲ度急性GVHD伴肺感染、巨细胞病毒血症、耐药铜绿假单胞菌败血症，最终于移植后14个月死于感染。

## 讨论

BCR::ABL1融合了22号染色体的BCR基因序列和9号染色体上的ABL1区域，形成具有酪氨酸激酶活性的BCR-ABL融合蛋白和特征性的费城染色体（Ph染色体）。在慢性髓性白血病（CML）中，Ph染色体可出现在95％以上的患者中，在成人急性淋巴细胞白血病（ALL）中占11％～29％，儿童ALL中仅为1％～3％，而在AML中发生率低于1.5％[4],[6],[10]–[11]。2016年WHO首次将AML伴BCR::ABL1划分为急性髓系肿瘤的一类新亚型，诊断要点包括无CML病史，不符合混合表型急性白血病诊断标准，不伴有其他重现性遗传学异常的AML，后续2022年的WHO分型继续沿用了该亚型的定义[1]–[2]。AML伴BCR::ABL1为一类罕见的白血病亚型，其诊断标准并不统一[12]–[13]。尤其AML伴BCR::ABL1同CML在细胞遗传学上十分相似，与急性期起病的CML急髓变（CML-MBC）很难鉴别。满足基本诊断要点的同时，需进一步从外周血及骨髓细胞形态学特点、脾大程度、分子生物学特征、疾病预后及治疗转归等方面逐步分析，从而鉴别诊断。但是以上特征并非绝对只存在于AML伴BCR::ABL1或CML-MBC，有无CML慢性期及加速期病史的临床过程是鉴别诊断两类疾病的关键[4]。

从外周血嗜碱性粒细胞比例来讲，AML伴BCR::ABL1患者外周血可见嗜碱性粒细胞，但比例<2％，CML-MBC≥2％。本组患者外周血嗜碱性粒细胞特点符合AML伴BCR::ABL1。骨髓细胞形态学方面，两者诊断上均有骨髓髓系原始细胞>20％的基本要求，但AML伴BCR::ABL1的髓系细胞以单一原始细胞为主，CML-MBC除了原始髓系细胞，通常还伴随中性中幼粒、晚幼及杆状核粒细胞等各阶段髓系细胞的明显增多[14]。初诊时本组患者骨髓形态学特征均表现为增生活跃，且以形态单一异常原始细胞为主。

AML伴BCR::ABL1患者脾脏多正常或轻中度肿大，CML-MBC患者多表现为脾脏重度肿大[15]。7例患者中仅1例伴重度脾大，同时综合其他鉴别诊断要点（包括外周血、骨髓细胞形态、既往病史、分子生物学及遗传学特征），仍考虑患者诊断AML伴BCR::ABL1。

BCR::ABL1基因在AML伴BCR::ABL1患者可以为P210或P190阳性，CML-MBC多为P210阳性。ABL激酶区突变在AML伴BCR::ABL1不易检测到，而在CML-MBC中易检测到[6]。该组患者均检测到BCR::ABL1阳性，其中6例为P210阳性，1例为P210、P190阳性，1例为P230阳性。并且7例患者在诱导（再诱导）化疗、巩固化疗及allo-HSCT后均未发现ABL激酶突变。对于两类疾病伴随基因突变在辅助鉴别诊断中的作用，尚无统一观点。有研究者认为，AML伴BCR::ABL1患者可伴随多种AML相关的突变，而在CML-MBC中则并不常见[16]。Zhou等[4]在15例AML伴BCR::ABL1中发现6例伴IDH1/IDH2突变，4例伴ASXL1突变，3例伴DNMT3A突变。Konoplev等[6]对9例AML伴BCR::ABL1患者行14种基因突变检测，发现2例患者出现NPM1突变[17]。还有研究者提出，抗原受体基因（IgH、TCR）、IKZF1和（或）CDKN2A的缺失支持AML伴BCR::ABL1的诊断，这些突变在CML-MBC中未见[11]。上述不同结果的出现，可能与AML伴BCR::ABL1这一疾病类型发生率低、缺乏大系列研究数据有关，仍需今后进一步研究明确。本研究7例患者检出4种基因突变，其中NPM1、RUNX1、ASXL1多见于AML，而并无IDH1/2突变数据。

以往研究显示单纯t（9;22）（q34;q11.2）是AML伴BCR::ABL1患者中较常见的细胞遗传学异常，此外还伴随+19、+8、+21、i（17q）和7号染色体异常，而出现非t（9;22）改变的细胞遗传学异常被认为是预后不良因素[6],[18]。本组7例患者中4例（57.1％）单纯存在t（9;22）（q34;q11.2），例3和例5伴有+8、i（17q），例4伴+8、+19、i（17q）。以上细胞遗传学异常未显示同预后相关，可能与病例数较少且均接受了allo-HSCT有关。

AML伴BCR::ABL1传统药物诱导化疗的缓解率为58.3％，联合TKI后缓解率明显提高，但是不能获得长期缓解[19]。目前对该类疾病尚无统一治疗方案，在2022 ELN关于白血病诊断及预后分组的建议中，该类患者对传统化疗耐受性差，归属于高危组，治疗上建议联合使用TKI[3]。对于缓解后治疗，多数学者认为应尽早行allo-HSCT，化疗+TKI巩固治疗的远期效果欠佳，但还有待进一步大样本的研究明确[20]。与AML伴BCR::ABL1类似，CML-MBC亦对传统化疗反应差，建议TKI联合化疗，并且获得血液学缓解后尽早进行allo-HSCT[21]。有研究认为BCR::ABL1不能作为预后不良的决定因素，其中大多数病例存在其他高风险细胞遗传学或分子学特征是其预后欠佳的关键[22]。虽然该类患者对传统化疗效果欠佳，桥接allo-HSCT后预后明显改善。Mizuno等认为接受allo-HSCT的AML伴BCR::ABL1患者的无病生存率与中危AML患者（非伴BCR::ABL1）相当，并且复发率明显低于中危/高危组，移植后预后优于高危组AML[20]。所以，TKI联合传统化疗，缓解后尽快行allo-HSCT可能有助于使AML伴BCR::ABL1患者的预后改善。本研究7例患者诱导化疗均联合TKI，缓解率为71.4％（5/7），高于传统药物化疗[19]。3例未获得CMR的患者allo-HSCT后3个月均获得CMR，6例患者长期生存，1例死于移植后并发症，3年OS率为（80.0±17.9）％。

然而，白血病复发仍是目前allo-HSCT后AML患者面临的一个挑战。越来越多的研究发现，移植后的维持治疗包括MRD阳性抢先治疗和缓解后预防治疗可使患者获益[23]。目前无关于AML伴BCR::ABL1患者allo-HSCT后维持治疗的相关报道。本组中心6例患者于移植后4（1～6）个月开始维持治疗。例1因移植后P210阳性，于移植后1个月加用达沙替尼抢先治疗，移植后3个月融合基因转阴，但该患者因后续发生重度GVHD、重症感染，于移植后14个月死亡。余5例患者于移植后3～6个月期间加用TKI治疗（其中2例患者因药物不良反应调整药物种类），维持治疗时间为1～1.5年。我们认为该组患者的的较高无复发生存率既与allo-HSCT提高CMR有关，也可能获益于TKI维持治疗。对于靶向药物在该类患者中的不良反应及耐药问题，仍需要进一步探讨。

本研究结果初步显示，诱导化疗联合TKI方案对于治疗AML伴BCR::ABL1患者具有较好疗效，桥接allo-HSCT可进一步提高CMR，移植后TKI维持治疗可能有助于改善预后。上述结论仍需要前瞻性、多中心、大样本的研究进一步验证。
